# A Frustrated Antipolar Phase Analogous to Classical Spin Liquids

**DOI:** 10.1002/adma.202410282

**Published:** 2024-10-23

**Authors:** Gaël Bastien, Dalibor Repček, Adam Eliáš, Andrej Kancko, Quentin Courtade, Tetiana Haidamak, Maxim Savinov, Viktor Bovtun, Martin Kempa, Karel Carva, Michal Vališka, Petr Doležal, Marie Kratochvílová, Sarah A. Barnett, Petr Proschek, Jan Prokleška, Christelle Kadlec, Petr Kužel, Ross H. Colman, Stanislav Kamba

**Affiliations:** ^1^ Department of Condensed Matter Physics, Faculty of Mathematics and Physics Charles University Ke Karlovu 5 Prague 2 121 16 Czech Republic; ^2^ Institute of Physics of the Czech Academy of Sciences Na Slovance 2 Prague 182 00 Czech Republic; ^3^ Department of Solid State Engineering, Faculty of Nuclear Sciences and Physical Engineering Czech Technical University in Prague Břehová 7 Prague 1 115 19 Czech Republic; ^4^ Diamond Light Source Chilton Didcot Oxfordshire OX11 0DE UK

**Keywords:** dielectric relaxation, electric dipoles, frustration, highly degenerate state, structural disorder

## Abstract

The study of magnetic frustration in classical spin systems is motivated by the prediction and discovery of classical spin liquid states. These uncommon magnetic phases are characterized by a massive degeneracy of their ground state implying a finite magnetic entropy at zero temperature. While the classical spin liquid state is originally predicted in the Ising triangular lattice antiferromagnet in 1950, this state has never been experimentally observed in any triangular magnets. The discovery of an electric analogue of classical spin liquids on a triangular lattice of uniaxial electric dipoles in EuAl_12_O_19_ is reported here. This new type of frustrated antipolar phase is characterized by a highly‐degenerate state at low temperature implying an absence of long‐range antiferroelectric order, despite short‐range antipolar correlations. Its dynamics are governed by a thermally activated process, slowing down upon cooling toward a complete freezing at zero temperature.

## Graphical abstract

EuAl_12_O_19_ crystals realize an electric analogue to one of the famous models of frustrated magnetism: the Ising triangular lattice antiferromagnet. Electric dipoles on a triangular lattice preferentially align antiparallel to their neighbors yet fail to form any long‐range order, even at the lowest temperatures. The resulting frustrated antipolar phase is analogous to classical spin liquids.

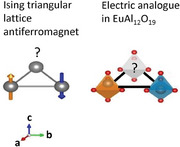

## 1 Introduction

**Figure 1:**
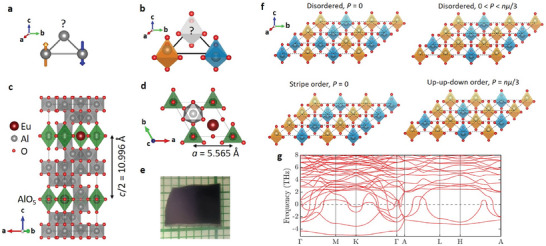
a) Frustrated magnetism in ITLAFM. It is impossible to place the three spins such that they all point in the opposite direction with respect to their neighbors. b) Triangle of Ising‐like electric dipoles realizing an equivalent frustration problem. The electric dipoles pointing toward ***c*** and toward ‐***c*** are colored in orange and blue, respectively and the displacement of the Al(5) ion is exaggerated for the clarity of the figure. c) View from the ***b*** ^*^ direction of the unit cell of the hexagonal crystal structure of EuAl_12_O_19_ at *T* = 35 K. The bipyramid AlO_5_ carrying electric dipoles are highlighted in green. d) View from the ***c*** axis of a selected part of the unit cell showing the triangular lattice of electric dipoles. e) Single crystal of EuAl_12_O_19_ on a millimeter‐paper. f) Examples of various electric dipole configurations at the minimum of energy of the ITLAFE. They are characterized by all the triangles harboring one or two dipoles pointing up, i.e., there is no triangle with three dipoles pointing in the same direction. Disordered configurations with zero polarization are favored at finite temperature by the minimization of the free energy. The stripe order and the up‐up‐down order are expected to be selected when the degeneracy is lifted by second nearest neighbor dipolar interactions and by external electric field, respectively.^[^ ^14^, ^15^, ^16^ ^]^ g) Calculated phonon dispersion curves for EuAl_12_O_19_ with the Al(5) ion sitting at the centre of the AlO_5_ bipyramid. Only phonons below 8 THz are shown to focus on the low energy branches. Imaginary frequencies are represented by negative values.

Frustrated magnets are systems where all the magnetic interactions cannot be simultaneously satisfied.^[^ ^1^, ^2^ ^]^ In classical spin systems, this frustration can lead to highly degenerate ground states, referred to as classical spin liquid states. These uncommon magnetic phases have been experimentally observed in pyrochlore magnets^[^ ^3^, ^4^ ^]^ and in Kagome magnets.^[^ ^5^ ^]^ The canonical historic example of a classical spin liquid is the type proposed by Wannier in 1950, in the Ising triangular lattice antiferromagnet (ITLAFM).^[^ ^6^ ^]^ This model consists of Ising spins arranged in a planar triangular lattice with antiferromagnetic interactions favoring antiparallel alignment of neighboring spins (**Figure** **1**a). There is not a single configurational state with lowest magnetic energy, instead many possible energy‐degenerate states result in the same lowest energy. This implies an absence of long‐range ordering and a finite magnetic entropy at zero temperature, in contradiction to the third law of thermodynamics.^[^ ^2^, ^4^, ^6^ ^]^ The disordered ground state is topological, since spin configurations can be described in terms of topologically protected strings, which can be formed or broken only upon nucleation of spinons.^[^ ^7^, ^8^ ^]^ However, this classical spin liquid state has never been observed in any ITLAFM. Indeed, experimental realizations of an *S* = 1/2 ITLAFM deviate from the strict Ising limit and are better described by the quantum Ising model, where transverse quantum spin fluctuations or an effective transverse field plays a major role.^[^ ^9^, ^10^, ^11^, ^12^, ^13^ ^]^

Several models initially developed to describe magnetism have already motivated the discovery and study of new phenomenon in dielectrics such as electric dipolar glasses^[^ ^17^, ^18^, ^19^ ^]^ and ferroelectric quantum criticality.^[^ ^20^, ^21^ ^]^ The discovery of geometrical frustration in ice crystals ^[^ ^22^, ^23^ ^]^ inspired the research on frustrated magnetism in spin ices^[^ ^2^, ^3^, ^4^ ^]^ and the research on ices of electric dipoles.^[^ ^24^, ^25^, ^26^, ^27^ ^]^ The study of quantum magnetism in organic triangular magnets revealed also the presence of a frustrated lattice of charge‐displacive electric dipoles, which may realize a quantum electric dipole liquid besides the putative quantum spin liquid state.^[^ ^28^, ^29^ ^]^

In this paper, we propose to explore frustrated magnetism of ITLAFMs based on the study of an analogue of the ITLAFM on a lattice of electric dipoles. Indeed, ion‐displacive electric dipoles have the advantage that they can be intrinsically Ising.^[^ ^21^, ^30^, ^31^ ^]^ The magnetoplumbite crystal structure in particular harbors a triangular lattice of Ising‐like electric dipoles formed by an MO_5_ bipyramid (M = Al, Ga, Fe)^[^ ^30^, ^31^, ^32^, ^33^, ^34^, ^35^, ^36^ ^]^ (Figure 1b–d). The combined effects of a triangular geometry, and interactions favoring antipolar arrangement of neighboring dipoles results in an electric frustration (Figure 1b).^[^ ^16^, ^37^ ^]^ Thus the magnetoplumbite crystal structure realizes an electric analog of the *S* = 1/2 ITLAFM that we propose to call the Ising triangular lattice antiferroelectric (ITLAFE).

The geometrical frustration of the magnetoplumbite crystal structure was first noted by Wang et al.^[^ ^16^ ^]^ Using DFT calculations, they showed that the interaction between electric dipoles is dominated by electric dipole‐dipole interactions. As a consequence they favor antipolar alignment of dipoles located on the same triangular layer, and polar alignment of electric dipoles on adjacent layers. However experimental and theoretical studies of barium‐hexaferrite BaFe_12_O_19_ revealed the important role of quantum fluctuations favoring a quantum paraelectric ground state.^[^ ^21^, ^31^, ^35^, ^37^, ^38^, ^39^ ^]^ One way to reduce the influence of quantum fluctuations on the dielectric properties is to change the ion in the double pyramid from Fe^3+^ to the smaller Al^3+^, as theoretically proposed by Wang et al.^[^ ^16^ ^]^ The presence of AlO_5_ electric dipoles in the hexaaluminates AAl_12_O_19_ (A = Ca, Sr, Pb) has previously been confirmed using X‐ray and neutron diffraction,^[^ ^32^, ^33^, ^40^ ^]^ however, low‐temperature dielectric studies of these materials have been lacking.

In this paper, we reveal that the lattice of electric dipoles in the hexaaluminate EuAl_12_O_19_ is an electric analogue of the classical frustrated spin systems. EuAl_12_O_19_ is a luminescent material^[^ ^41^, ^42^ ^]^ and a quasi‐2D ferromagnet below *T*_C_ = 1.3 K.^[^ ^43^ ^]^ We report the occurrence of a second order phase transition in EuAl_12_O_19_ at *T*_S_ = 49 K with a strong enhancement of the dielectric permittivity below this transition. Combining polarization measurements and single crystal synchroton X‐ray diffraction we show that this transition does not correspond to any ferroelectric or antiferroelectric ordering. The AlO_5_ electric dipoles build up short‐range antipolar correlations upon cooling but they remain dynamically disordered at all temperatures due to the geometrical frustration. We observe a soft dielectric relaxation coming from the electric dipoles and we have followed this relaxation from the THz range at room temperature down to the Hz range near *T* ≈ 5 K. The dynamic switching between degenerate dipole configurations is a thermally activated process well described by the Arrhenius law.

Based on our data we propose that the electric dipoles, created by the displacement of Al^3+^ ions within their AlO_5_ bipyramids, form a frustrated antipolar state. The electric dipoles build up short‐range correlations upon cooling but they do not form any long‐range order, thus the frustrated antipolar state is analogous to ordinary liquids, which lack crystalline order and to classical spin liquids, which lack magnetic order despite strong magnetic correlations.^[^ ^2^, ^3^, ^4^, ^5^ ^]^ Therefore, we propose to name it a classical electric dipole liquid. A few examples of ordered and disordered electric dipole configurations occupying the minimum of energy are given in Figure 1f. Disordered dipole configurations are favored by a large configurational entropy of *S* = 0.323*R*.^[^ ^6^ ^]^ The system evolves continuously among the manifold of degenerate states, with its dynamics slowing down upon cooling toward a complete freezing only at *T* = 0 K, as predicted for classical spin liquids.^[^ ^2^, ^3^, ^44^ ^]^ This classical electric dipole liquid state differs by its dynamical properties from electric dipolar glasses and spin glasses which occur in disordered systems and freeze at a finite temperature.^[^ ^17^, ^18^, ^19^ ^]^ It also differs from quantum electric dipole liquids and quantum spin liquid states which remain dynamic even at zero temperature due to quantum fluctuations.^[^ ^2^, ^28^, ^29^, ^38^ ^]^

## 2 Results and Discussion

### 2.1 Antipolar Interactions Indicated by Crystal Structure Relaxation and Phonon Spectrum Computation

We have examined theoretically the presence of interacting electric dipoles in EuAl_12_O_19_ by computing lattice vibration spectra using the DFT+U method (Figure 1g). The calculation performed with the Al(5) ion sitting at the center of the AlO_5_ bipyramid shows a large number of soft modes with imaginary frequencies throughout the Brillouin zone, plotted as negative frequencies in Figure 1g. It confirms the instability of the paraelectric crystal structure of EuAl_12_O_19_ described by the unit cell shown in Figure 1c (space group *P*6_3_/*mmc*). These results show similarity with the phonon calculation in isostructual BaFe_12_O_19_ indicating the same instability.^[^ ^16^, ^37^ ^]^ A second calculation was performed with the Al(5) ion in an off‐centered position implying a ferroelectric crystal structure (space group *P*6_3_ *mc*) (See Figure S1 and Table S1, Supporting Information). The two dipoles within the unit cell prefer parallel alignment, implying ferroelectric order along the ***c*** axis. Imaginary phonon frequencies were still observed approaching the boundary of the Brillouin zone in the planar direction indicating that phonon modes with Al(5) displaced oppositely in the neighboring cell would be of lowest energy. In order to verify the character of interactions between neighboring polar cells we have also evaluated energies of a supercell composed of two unit cells (along the ***a*** axis) with the two possible dipole alignments. We found that the antiparallel ordered system has energy lower than the parallel one by 26 meV per f.u. This interaction clearly prefers antipolar arrangement, and the same is expected along the ***b*** axis due to symmetry. Combined with the triangular geometry, these antipolar interactions imply the geometric frustration of the lattice of electric dipoles.

### 2.2 Unusual Phase Transition at *T*_*S*_ = 49 K

**Figure 2:**
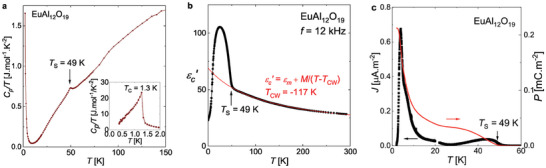
a) Specific heat divided by temperature *C*_*p*_/*T* of EuAl_12_O_19_ as a function of temperature revealing a second order phase transition at *T*_*S*_ = 49 K and the ferromagnetic transition at *T*_*C*_ = 1.3 K. b) Dielectric permittivity of EuAl_12_O_19_ along the ***c*** axis measured at a frequency of *f* = 12 kHz as a function of temperature. The transition at *T*_S_ =49 K is clearly indicated by an abrupt change of slope. The red curve is a Curie–Weiss fit giving the large negative Curie–Weiss temperature of *T*_CW_ = −117 K. c) Pyroelectric current in black and polarization in red as a function of temperature. The crystal was initially cooled down to *T* = 2 K under an electric field of *E*_DC_ = 3.2 kV cm^−1^ along the ***c*** axis. Then the pyroelectric current was measured upon heating at *q* = 1 K min^−1^ in absence of electric field. It shows a strong reduction of the polarization around *T* ≈ 4 K followed by its suppression around  *T*_S_= 49 K.

Specific heat measurements in EuAl_12_O_19_ show a succession of two second order phase transitions at *T*_S_ = 49 K and *T*_C_ = 1.3 K as represented in **Figure** **2**a. The latter one at *T*_C_ = 1.3 K was previously identified as a ferromagnetic ordering of magnetic moments on the Eu^2+^ site.^[^ ^43^ ^]^ The former transition at *T*_S_ = 49 K is accompanied by an abrupt change of the slope in the temperature dependence of the dielectric permittivity ɛ′ and the suppression of pyroelectric currents, as shown by Figure 2b,c. Such dielectric anomalies are usually associated with improper or pseudoproper ferroelectric transition.^[^ ^45^, ^46^, ^47^ ^]^

At temperature higher than *T*_S_ = 49 K, the dielectric permittivity ɛ′ follows the Curie–Weiss (C‐W) law: ɛ′ = ɛ_*m*_ + *M*/(*T* − *T*_CW_). The negative Curie–Weiss temperature *T*_CW_ = – 117 K indicates incipient ferroelectricity.^[^ ^20^, ^31^ ^]^ ɛ_*m*_ = 11.4 stands for the temperature independent contribution to ɛ′. The parameter *M* = 6750 K gives us an estimate of the magnitude of the electric dipoles *μ* = 0.97 *e*.Å  using the equation *M* = *n* *μ* ^2^/*k*_B_, where *n* is the density of electric dipoles and *k*_B_ is the Boltzmann constant.^[^ ^31^ ^]^ Below *T* = 15 K, ɛ′ drops much below the value given by the C‐W law indicating the shift of dielectric relaxation frequency below the measurement frequency. This result contrasts with the previously studied isostructural compounds SrFe_12_O_19_ and BaFe_12_O_19_, where strong quantum fluctuations prevent any slowing down of the dynamics of the electric dipoles at low temperature.^[^ ^21^, ^31^, ^35^, ^37^ ^]^

To test whether the transition at *T*_S_ = 49 K in EuAl_12_O_19_ is a ferroelectric transition, we probed the electrical polarization both with second harmonic generation (SHG) experiments and pyroelectric current methods. SHG was measured both along and perpendicular to the crystallographic ***c*** axis in the temperature range from 10 K to room temperature. No SHG signal was observed neither above or below *T*_S_ = 49 K confirming that the structure remains centrosymmetric. Measurements of pyroelectric current indicate a weak polarization in poled EuAl_12_O_19_ below *T*_S_ = 49 K (Figure 2c). However, it grows linearly with the electric field applied upon cooling without any hint of saturation up to *E*_DC_ = 6 kV cm^−1^ and it can be interpreted as an electric‐field induced polarization $P=\varepsilon_{0}\varepsilon_{c}^{\prime }E$ (see Figure S2, Supporting Information). Thus SHG experiment and the pyroelectric current experiments both show that the centre of symmetry of EuAl_12_O_19_ is preserved at all temperatures in the absence of an external electric field and the transition at *T*_S_ = 49 K is not a ferroelectric ordering transition. The observation of a polarization measured after cooling under electric field can be explained by the preferential selection of dipole configurations from the ground‐state manifold that have finite polarization (see Figure 1f). The temperature dependence of the electrical polarization is rather unusual since we observe its strong reduction above *T* = 4 K (Figure 2c), which is not directly related to any phase transition. This feature can be interpreted as a dynamical release of the polarization and it will be discussed in more detail later in the context of the relaxation processes detected by THz, microwave and dielectric spectroscopies.

The strong increase of ɛ′ below *T*_S_ = 49 K could in principle come from the contribution of ferroelectric domain walls like in the improper ferroelectric 2*H*‐BaMnO_3_.^[^ ^47^ ^]^ We excluded this scenario by the measurement of an independence of ɛ′ on applied static (DC) electric field, at least up to *E*_DC_ = 4.5 kV cm^−1^ (see Figure S3, Supporting Information).^[^ ^47^, ^48^, ^49^ ^]^ This result further confirms the absence of ferroelectric order in EuAl_12_O_19_.

### 2.3 Absence of Symmetry Change at *T*_S_ = 49 K from X‐Ray Diffraction

To precisely map out structural changes induced upon cooling in EuAl_12_O_19_, we performed single crystal X‐ray diffraction at temperature down to *T* = 35 K. On cooling from 225 to 35 K no signatures of any symmetry change, such as the appearance of superstructure peaks, is evident (see Figure S4 and Table S II–S V, Supporting Information). The best space group to describe the diffraction results remains *P*6_3_/*mmc* at all temperatures. All reflection extinction conditions of the *P*6_3_/*mmc* space group are strictly maintained within the resolution of our data down to the lowest measured temperature and attempts to fit with lower symmetry space group such as the ferroelectric space group *P*6_3_ *mc* or the centrosymmetric space group $P3\bar{m}1$ did not lead to any significant improvement of the refinement. These measurements indicate that the electric dipole lattice is disordered at all temperatures. As a consequence the transition at *T*_S_ = 49 K does not correspond to the formation of the antiferroelectric stripe order previously predicted by Monte Carlo calculations^[^ ^16^ ^]^ and the nature of this transition remains unclear. The distance between the equilibrium position of Al(5) and the center of the bipyramid at *T* = 225 K was estimated at *δ* = 0.21 Å  from structural refinement. The room temperature off‐centering displacement extracted from X‐ray or neutron diffraction in materials with the magnetoplumbite increases slightly with the ionic radius of the A^2+^ ion from *δ* = 0.17 Å  in CaAl_12_O_19_ to *δ* = 0.21 Å^[^ ^32^, ^33^, ^40^ ^]^ in EuAl_12_O_19_ and it decreases with the ionic radius of the B^3+^ ion from *δ* = 0.21 Å  in EuAl_12_O_19_ and SrAl_12_O_19_ to *δ* = 0.10 Å  in SrFe_12_O_19_.^[^ ^32^, ^40^, ^50^ ^]^ While no significant evolution of the off‐centering with temperature were measured in hexaaluminates EuAl_12_O_19_ and CaAl_12_O_19_,^[^ ^40^ ^]^ the off‐centering of the Fe^3+^ ion in BaFe_12_O_19_ gets significantly reduced upon cooling from *δ* = 0.177 Å  at *T* = 295 K down to *δ* = 0.098 Å  at *T* = 4 K.^[^ ^34^, ^35^ ^]^ The increased separation distance at low temperature results in a larger barrier to quantum tunneling of the ion and it explains why EuAl_12_O_19_ is much less sensitive to quantum fluctuations than the previously studied BaFe_12_O_19_.^[^ ^37^, ^38^ ^]^

**Figure 3:**
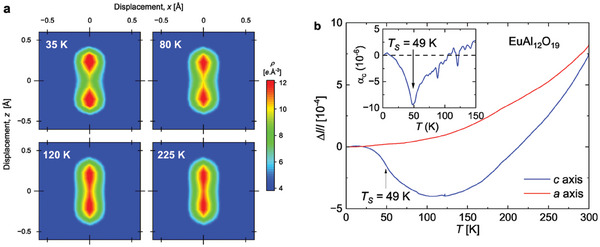
a) Temperature dependence of the electron density distribution obtained from MEM analysis confirming that the Al(5) ion is trapped in a double well potential. The figure consists in 2D‐cuts within the (101) plane, centered at (0,0,1/4). b) Relative length change of EuAl_12_O_19_ along the *a* and ***c*** axis as a function of temperature. The inset shows the thermal expansion coefficient *α*_*c*_ as a function of temperature.

To more clearly follow the temperature dependence of the positional distribution of the Al(5) ion within its double pyramid, we performed maximum‐entropy method (MEM) analysis on the phased single crystal synchrotron structure factor data. (**Figure** **3**a; Figure S5 and Figure S6, Supporting Information). The electron density distribution confirms a double well potential of the Al(5) site, with a distance between the equilibrium position and the center of the bipyramid at *T* = 35 K of *δ* = 0.24 Å  in agreement with *δ* = 0.22 Å  from the structural refinement.

We also observed negative thermal expansion along the ***c*** axis within a broad range of temperature from 12 to 110 K both by thermal expansion measurements (Figure 3b) and by single crystal X‐ray diffraction (Figure S7, Supporting Information). The thermal expansion coefficient *α*_*c*_ undergoes a minimum around the transition at *T*_S_ = 49 K. This negative thermal expansion must come from the slowing down of the vibration of the Al(5) ion in the double pyramid^[^ ^51^ ^]^ and from the building up of antipolar correlations between electric dipoles.^[^ ^52^, ^53^ ^]^ Thus the observation of negative thermal expansion across a broad temperature range indicates that the formation of antipolar correlations between electric dipoles upon cooling is a continuous process, like the formation of magnetic correlations in frustrated magnets.^[^ ^1^, ^16^, ^52^ ^]^

### 2.4 Dynamical Properties of the Lattice of Electric Dipoles

**Figure 4:**
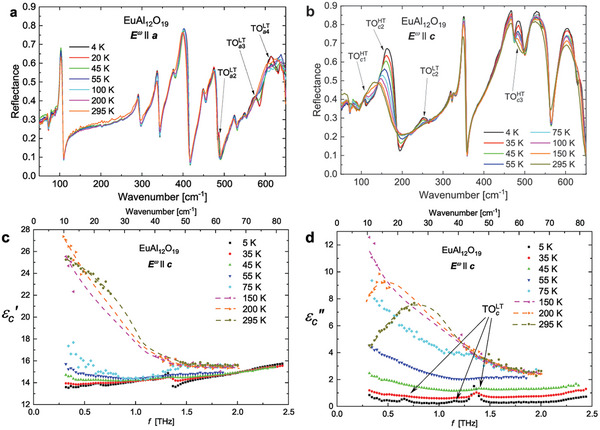
a,b) Temperature evolution of the IR reflectivity spectra of EuAl_12_O_19_ measured in the ***E*** ^*ω*^∥ ***a*** configuration   and in the ***E*** ^*ω*^∥ ***c*** configuration, respectively. New transverse optic modes get visible upon cooling, they are pointed by arrows and labeled ${\mathrm{TO}}_{a}^{\mathrm{LT}}$ for ***E*** ^ω^∥ ***a*** and ${\mathrm{TO}}_{c}^{\mathrm{LT}}$ for ***E*** ^ω^∥ ***c***. Some other modes already visible at room temperature get strongly enhanced upon cooling, they are labelled ${\mathrm{TO}}_{c}^{\mathrm{HT}}$. c,d) Frequency dependence of the THz real **c** and imaginary parts **d** of the complex permittivity spectra of EuAl_12_O_19_, measured in the ***E*** ^ω^∥ ***c*** configuration. A broad relaxation R1 spreads practically across the whole THz range at *T* = 295 K and its absorption is so strong that there is even missing gap in the original transmittance data, filled by dashed line as guide to the eye. ${\mathrm{TO}}_{c}^{\mathrm{LT}}$ indicates three phonon modes appearing below *T*_S_ = 49 K.

We have investigated the dynamical properties of the electric dipoles by probing phonons and dielectric relaxations in EuAl_12_O_19_ over 13 orders of magnitude of frequencies from infrared (IR) frequencies (≈10^13^ Hz) down to 1 Hz. The spectra of IR reflectivity for ***E*** ^*ω*^∥ ***a*** and ***E*** ^*ω*^∥ ***c*** reveal the appearance and reinforcement upon cooling of several IR active modes, without any sudden changes at *T*_S_ = 49∼K (see **Figure** **4**a,b). This evolution must be related to changes of local symmetry induced by the formation of short range antipolar correlations. Nevertheless, the THz complex permittivity directly calculated from the measured complex transmittance spectra reveal a broad dielectric relaxation which slows down on cooling for ***E*** ^*ω*^∥ ***c*** (see Figure 4c,d; see also Figure S8, Supporting Information, for the behavior of the complex permittivity for ***E*** ^*ω*^∥ ***a***). The excitation behaves like an overdamped oscillator, resembling the behavior of a dielectric relaxation,^[^ ^54^ ^]^ and therefore we call it relaxation R1. The softening of its frequency *f*_R1_ quantitatively explains the C‐W behavior of ɛ′ below *f* = 1.8 GHz because the product Δɛ_R1_ *f*_R1_ should be temperature independent (as Δɛ_R1_ marks the contribution of R1 to ɛ′) and because the sum of phonon contributions in ɛ′ (obtained from the fits of IR reflectivity using a Lorentz three‐parameter oscillator model)^[^ ^54^ ^]^ slightly decreases on cooling due to hardening of the ${\mathrm{TO}}_{c}^{\mathrm{HT}}$ mode (Figure 4b). We propose that the microscopic origin of the R1 relaxation is connected to the dynamical disorder of the Al(5) cations within the AlO_5_ bipyramids.

**Figure 5:**
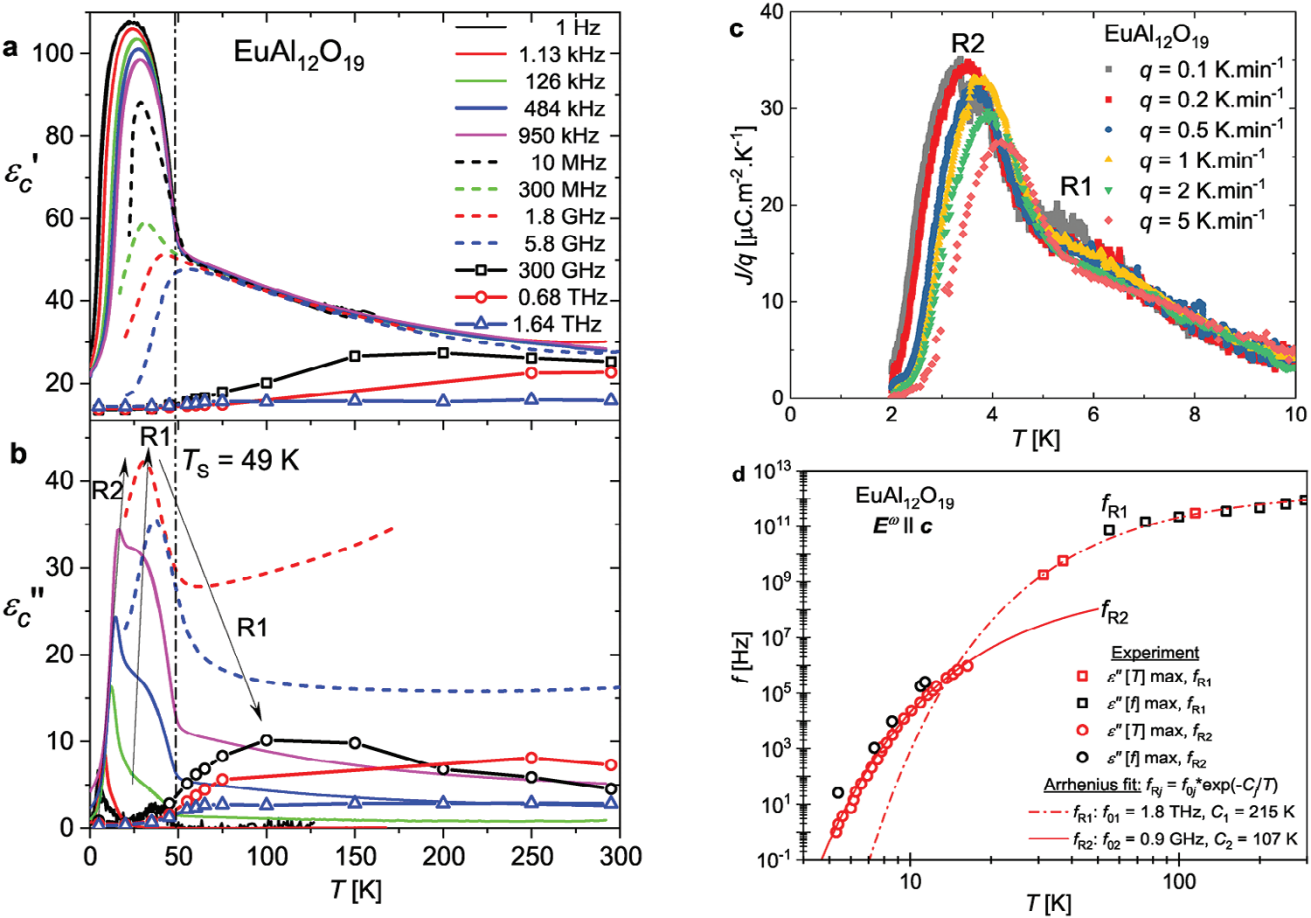
Temperature dependence of a) the real part $\varepsilon_{c}^{\prime }$ and b) the imaginary part $\varepsilon_{c}^{\prime \prime }$ of the dielectric permittivity at various frequencies. The relaxations R1 and R2 are indicated by a broad maximum and a sharp peak in $\varepsilon_{c}^{\prime \prime }\left(T\right)$. c) Pyroelectric currents density divided by the heating rate *q* for different heating rates. The crystal was cooled under an electric field of *E* = 3.2 kV cm^−1^ along the ***c*** axis. d) Temperature dependence of the *f*_R1_ and *f*_R2_ relaxation frequencies obtained as positions of maxima from either $\varepsilon_{c}^{\prime \prime }\left(f\right)$ measured at a particular temperature or $\varepsilon_{c}^{\prime \prime }\left(T\right)$ spectra measured at a particular frequency. The lines are two Arrhenius fits.

To follow the softening of the relaxation R1 upon cooling, we extended the measurement of complex permittivity down to the microwave (MHz‐GHz) and low frequency (Hz‐kHz) range (**Figure** **5**a,b). The frequency of the relaxation R1 corresponds to the frequency where the maximum of $\varepsilon_{c}^{\prime \prime }\left(\;f\right)$ occurs. Its softening is remarkable, since *f*_R1_ steadily decreases from ≈0.9 THz at 295 K to about 1 kHz at 10 K. In the Hz‐kHz range $\varepsilon_{c}^{\prime \prime }\left(T\right)$ exhibits a sharp peak ascribed to a second excitation of the relaxational type R2 and the maximum of the broad bump associated with the relaxation R1 is not fully discernible (Figure 5b). The R2 relaxation appears only below the transition *T*_S_ = 49 K explaining the sudden change of slope of $\varepsilon_{c}^{\prime }\left(T\right)$ at this transition for frequencies below *f* ≈ 300 MHz. It indicates that this transition opens a new route for the flipping of the electric dipoles. These dielectric permittivity measurements were complemented by measurements of the elastic constant *C*_11_ and *C*_33_ from sound velocity experiments (See Figure S9, Supporting Information). They also reveal an anomalous hardening along the ***c*** axis below the phase transition at *T*_S_ = 49 K.

The temperature dependence of the *f*_R1_ frequencies in the GHz‐THz range and of the *f*_R2_ frequencies in the Hz‐MHz range are well fitted by Arrhenius laws (Figure 5d) with the activation energies *C*_1_ = 215 K and *C*_2_ = 107 K for R1 and R2, respectively. Note that the Vogel–Fulcher law describing freezing of a dielectric relaxation (for example in relaxor ferroelectrics and dipolar glasses)^[^ ^17^, ^18^, ^19^, ^54^ ^]^ could not better describe the data. In addition, attempts to model the data with the generalized Cochran formula *f*_R1_ = *A*(*T* − T*) describing slowing‐down of critical relaxation approaching a critical temperature T* could also not appropriately fit our data and therefore cannot explain the phase transition at *T*_S_. The Arrhenius behavior confirms that the dynamics of the electric dipoles is mainly thermally induced^[^ ^55^ ^]^ and it implies an asymptotic freezing at *T* = 0 K, as expected for a classical liquid of either spins or electric dipoles.^[^ ^2^, ^3^, ^44^ ^]^

The R1 and R2 relaxations can also help us to understand the unusual shape of the temperature dependence of the pyroelectric currents below 10 K. Indeed, the pyroelectric current measurements show a peak around 4 K shifting to higher temperature upon increasing the heating rate (Figure 5c). This rate‐dependent shift confirms that it corresponds to a dynamical release of polarization and not to a phase transition.^[^ ^56^, ^57^ ^]^ The peak of polarization current is composed of a main peak around 4 K and a broad shoulder around 6 K, which can be ascribed to R2 and R1 relaxations, respectively. Thus, the measurements of pyroelectric currents allow us to follow qualitatively the relaxation frequencies *f*_R1_ and *f*_R2_ up to longer time scales given by heating rate (*t* ≈ 10 − 100 s), analogous to the use of thermoremanent magnetization measurements of slow dynamics in frustrated magnets.^[^ ^58^, ^59^ ^]^

### 2.5 Synthesis of the Results and Interpretation

Altogether our measurements enable us to draw the general picture of the behavior of the lattice of electric dipoles in EuAl_12_O_19_. Around room temperature, the Al(5) jump between two potential minima in the AlO_5_ bipyramids due to thermal fluctuations described by the relaxation R1. Upon cooling antipolar correlations between neighboring electric dipoles get reinforced and the classical electric dipole liquid forms. The R2 relaxation appearing below *T*_S_ = 49 K can be ascribed to correlated flipping of electric dipoles. Both relaxation R1 and R2 soften upon cooling such that their characteristic time 1/*f*_R1_ and 1/*f*_R2_ diverge toward *T* = 0 K implying a nearly static disorder at low temperature. Following theoretical description of the excitations in the ITLAFM model,^[^ ^7^, ^8^ ^]^ the relaxation R2 can be attributed to the displacement of topologically protected objects such as topological strings and the electric equivalent of spinons.^[^ ^7^, ^8^ ^]^ Compared to spin ices and ices of electric dipoles, where classical spin liquid states and their electric analogues were previously reported,^[^ ^2^, ^3^, ^4^, ^24^, ^25^, ^26^ ^]^ the highly degenerate ground state of the ITLAFM and ITLAFE includes states with finite magnetization and polarization, respectively. This implies the possibility to record information locally by cooling under applied electric field^[^ ^15^ ^]^ as demonstrated in our pyroelectric current measurements (Figure 2c; Figure S1, Supporting Information).

In real materials, we expect the degeneracy of the ground state of the ITLAFM and ITLAFE to be lifted by second nearest neighbor interactions. Together with interplane interactions they favor the formation of a long‐ range 3D order, implying a collapse of the entropy and the subsequent fulfilment of the third law of thermodynamics.^[^ ^14^, ^16^, ^60^ ^]^ Especially, the classical Monte Carlo simulations performed by Wang et al.^[^ ^16^ ^]^ predicted the occurrence of a phase transition at *T* = 3 K for BaFe_12_O_19_, where the electric dipole liquid state would crystallize into the stripe antiferroelectric order (depicted in Figure 1f ). The true ground state of EuAl_12_O_19_ may also be the antiferroelectric stripe order due to a lifting of the high degeneracy of the electric dipole liquid state by second nearest neighbor dipolar interactions. Since dielectric permittivity and pyro‐electric currents measurements do not show any signature of antiferroelectric transition down to 0.3 K, the formation of the long‐range antiferroelectric order is certainly avoided by a form of kinetic arrest, the divergence of the timescale of the dynamics of the electric dipoles toward *T* = 0 K.

## 3 Conclusion

The study of the dielectric and structural properties of the compound EuAl_12_O_19_ leads to the discovery of a frustrated antipolar phase. The frustration arises from a triangular lattice of uniaxial electric dipoles coupled by interactions favoring antipolar ordering of nearest neighbors. The electric dipoles form short‐range correlations upon cooling, they remain disordered at any temperature and they do not show any conventional freezing at finite temperature. This behavior is similar to the one of the predicted classical spin liquid state in Ising triangular lattice antiferromagnets (ITLAFM). However, in contrast to the ITLAFM model, EuAl_12_O_19_ harbors a second order phase transition at *T*_S_ = 49 K showing additional complexity in the electric analogue EuAl_12_O_19_. Further investigations of the nature of this transition will be needed for a better understanding of the electric dipole frustration.

The discovery of this unusual dielectric phase will motivate further investigations of the dielectric properties of the broad variety of compounds harboring the magnetoplumbite crystal structure.^[^ ^32^, ^40^, ^41^, ^61^, ^62^ ^]^ It will also promote further work on electric dipole frustration in the pyrochlore structure^[^ ^24^, ^25^, ^26^, ^27^ ^]^ and it will motivate the search for electric analogues of other class of geometrically frustrated magnets such as Kagome magnet and Kitaev magnets.^[^ ^2^ ^]^ The classical electric dipole liquid state revealed in EuAl_12_O_19_ could be a good precursor for the formation of its quantum analogue, the quantum electric dipole liquid state, which could be valuable for quantum computing due to the presence of long‐range quantum entanglement.^[^ ^28^, ^29^, ^38^ ^]^

## 4 Experimental Section

### Density Functional Theory (DFT) Calculations

DFT+U calculations employed the full‐potential linear augmented plane wave (FP‐LAPW) method in the band structure program ELK,^[^ ^63^ ^]^ combined with the generalized gradient approximation (GGA) parameterized by Perdew–Burke–Ernzerhof.^[^ ^64^ ^]^ These calculations have used the Hubbard *U* = 2.7 eV applied to Eu 4*f* orbitals which was added to the Hamiltonian within the fully localized limit with double counting treatment.^[^ ^65^ ^]^ The full Brillouin zone was sampled by 50 *k*‐points, which is found to be sufficient for system with such large unit cell. The maximum length of the reciprocal lattice vectors for expanding the interstitial density and potential was enhanced to 20 to correctly treat the situation with a large mismatch between different atom muffin‐tin radii. The crystal structure obtained from X‐ray diffraction was relaxed prior to phonon calculations with and without maintaining the Al(5) in the *z* = 1/4 plane. Phonon dynamical matrix elements were obtained from forces calculated self‐consistently in supercells generated by the code PHONOPY.^[^ ^66^ ^]^

### Crystal Growth and Preparation

Single crystals of EuAl_12_O_19_ were grown by the optical floating zone method from sintered polycrystalline rod. The polycrystalline rod was prepared from Al_2_O_3_ and Eu_2_O_3_ binary oxides, intimately mixed by grinding. The homogeneous mixture was packed into a latex mold under vibration, followed by hydrostatic pressing (2 tonnes for 15 min) to increase density. The pressed rods, of approximately 6 mm diameter, were then sintered in air at 1473 K for 24 h. Growth was achieved in an optical floating zone furnace (FZ‐T‐4000‐VI‐VPM‐PC), under a flowing atmosphere of 5% H_2_ in Ar (0.2 l min^−1^), with counter rotation of upper and lower rods (50 rpm). After slowly heating to the melting point, and stabilizing the molten zone, the crystal was grown at a constant pulling speed of 3 mm h^−1^, with minor adjustments to furnace power during the growth to maintain a stable molten zone. A single grain of about 5 mm × 40 mm × 3 mm was successfully obtained. This crystal was oriented by backscattering Laue diffraction. It was cleaved along the easy cleavage plane *ab* or cut by a wire saw in other directions.

### Specific Heat

Specific heat measurements were performed by the relaxation method using the PPMS from Quantum Design both with and without the ^3^He insert.

### Dielectric Permitivitty

The low frequency (1 Hz–1 MHz) dielectric response was measured on a parallel plate (with a thickness of 305 µm and an area of about 28 mm^2^) sample with gold electrodes using a Novocontrol Alpha‐AN high performance impedance analyzer. A helium cryostat equipped with a ^3^He refrigerator was used to measure from room temperature down to 0.4 K). The absolute value of the Hz‐MHz $\varepsilon_{c}^{\prime \prime }$ may include a slowly varying background in the whole temperature range caused by rather long supply cables needed for experiments at very low temperatures. Measurements under applied electric fields were performed with the same setup but only down to 8 K using Janis cryostat.

The dielectric measurement in the high‐frequency range (1 MHz – 1.8 GHz) was carried out with an Agilent 4291B impedance analyzer, a Novocontrol BDS 2100 coaxial sample cell and a Janis closed‐cycle He cryostat. The sample was polished to a cylinder (height 2.2 mm, diameter 1.92 mm) with the crystallographic ***c*** axis along the main axis, and with gold electrodes sputtered on the bases. Complex permittivity was calculated from measured complex impedance.

The microwave response at 5.8 GHz was measured using the composite dielectric resonator method.^[^ ^67^, ^68^ ^]^ The TE_01δ_ resonance frequency, quality factor and insertion loss of the base cylindrical dielectric resonator with and without the sample were recorded during heating from 10 to 400 K with a temperature rate of 0.5 K min^−1^ in a Janis closed‐cycle He cryostat. A sample (6.6 × 5.2 mm plate, 2 mm thick) was used without electrodes. Both *a* and *c* axes were in the plane. The sample was placed on top of the base dielectric resonator. The resonators were measured in the cylindrical shielding cavity using the transmission setup with a weak coupling by an Agilent E8364B network analyzer. The complex permittivity of the sample corresponds to in‐plane average and was calculated from the acquired resonance frequencies and quality factors of the base and composite resonators.

### Second Harmonic Generation (SHG)

The temperature dependence of the SHG signal was obtained using an optical setup powered by a Ti:sapphire femtosecond laser amplifier (Spitfire ACE) producing 40 fs long pulses with a center wavelength of 800 nm and repetition rate of 5 kHz. The sample was placed in a continuous‐He‐flow cryostat Oxford Instruments with glass windows and illuminated by a collimated polarized beam with a diameter of 1.5–2.0 mm and a pulse fluence up to 5 mJ cm^−2^ to verify the absence of SHG in the low‐temperature phase. The resulting signal generated in a transmission configuration at 400 nm was spectrally filtered, detected by an avalanche photodiode, and amplified by a lock‐in amplifier (MFLI, Zürich Instruments), which ensures a background noise suppression. Schema of the SHG experimental setup is available in Supporting Information of ref. [49]. Independent transmission measurements in the optical region confirmed that the sample was transparent up to 350 nm, thus the expected SHG signal at 400 nm should be well measurable.

### Pyroelectric Current Measurements

The pyroelectric currents were measured on a (001) oriented crystal with a thickness of 305 µm and an area of about 28 mm^2^ with gold electrodes. The crystal was cooled under electric field applied along the ***c*** axis down to *T* = 1.8 K. Then the electric field was removed and after a dwelling time of 15 min, the pyrroelectric currents were measured upon heating. The PPMS from Quantum Design was used to cool and heat the sample with stable rates 0.1–5.0 K min^−1^. The electrical current was measured by a Keithley 617 electrometer. A homemade insert was used to apply voltage up to 180 V on the crystal.

### Single Crystal X‐Ray Diffraction

Single‐crystal X‐ray diffraction was performed at the I19 beamline (Hutch 1) of the Diamond Light Source synchrotron in Harwell Oxford, UK.^[^ ^69^ ^]^ The wavelength used is the Zr edge, specifically 17.9976 keV (λ = 0.68890 Å). We used the three‐circle diffractometer equipped with a Pilatus 2M detector. Cooling of the sample was carried out using the Oxford Cryosystems N‐Helix. A crystal with approximate dimensions 60 × 60 × 10 µm^3^ was selected for the temperature‐dependent measurement. Diffraction data were collected at 35, 80, 120, and 225 K. Data processing (peak hunting, indexing, scaling, absorption correction) was performed using the Xia2‐DIALS pipeline.^[^ ^70^, ^71^, ^72^ ^]^ The output reflection file was used for structure solution and refinement in Jana2020,^[^ ^73^ ^]^ utilizing the charge flipping method via the built‐in software SuperFlip.^[^ ^74^ ^]^ At all temperatures, extinction conditions indicated the *P*6_3_/*mmc* space‐group as best match. The phased structure factor information, output from Jana2020 after solution in the *P*6_3_/*mmc* space‐group, was used to calculate the electron density distribution by maximum entropy method (MEM) via the Dysnomia software suite.^[^ ^75^ ^]^ A map splitting the unit cell into 72 × 72 × 288 voxels, with a flat starting electron density distribution was used. No observable difference was seen when starting from a density distribution model based on the solved crystal structure. The presented crystal structure and electron density map visuals were created using VESTA.^[^ ^76^ ^]^ The temperature dependence of the cell parameters (Figure S7, Supporting Information) was measured in a *θ*−*θ* diffractometer Siemens D500 equipped by a linear detector Mythen 1 K and a Cu X‐ray tube with Cu‐Kα_1, 2_ radiation. The reciprocal space mapping of selected diffraction maxima (0 0 26) and (6 0 0) were used. The sample was cooled down to 3 K inside a cryostat (ColdEdge) equipped with a piezorotator allowing the alignment of the sample in *ϕ* direction.

### Thermal Expansion

High‐resolution length changes along the ***a*** and ***c*** directions were measured using a miniature capacitance dilatometer^[^ ^77^ ^]^ connected to the AH2500A capacitance bridge implemented in PPMS from Quantum Design.

### Infrared (IR) Spectroscopy

Low‐temperature IR reflectance measurements in the frequency range 30−670 cm^−1^ (1‐20 THz) were performed using a Bruker IFS‐113v Fourier‐transform IR spectrometer equipped with a liquid‐He‐cooled Si bolometer (1.6 K) serving as a detector. IR measurements were performed on the polished, over 500 µm thick, plate‐like sample with crystallographic axes ***a*** and ***c*** oriented parallel to plate edges. Thus prepared sample was attached to a 4‐mm aperture and oriented in the desired way with respect to the polarized IR radiation. A continuous‐He‐flow cryostat Optistat with polyethylene windows was used for temperature control.

### Terahertz Spectroscopy

Temperature‐dependent THz spectra of complex transmittance between 5 and 85 cm^−1^ were obtained using a custom‐made time‐domain spectrometer utilizing a Ti:sapphire femtosecond laser. Complex dielectric spectra were calculated directly from the complex transmittance spectra. The measurements were performed on a polished, free‐standing, planeparallel, 509 µm thick, plate‐like sample with crystallographic axes ***a*** and ***c*** oriented parallel to plate edges. The sample was attached to a 2.5‐mm aperture and oriented in the desired way with respect to the polarized THz radiation. A continuous‐He‐flow cryostat Oxford Instruments with mylar windows was used for temperature control. Details of the THz spectrometer and the principle of time‐domain THz measurement are described elsewhere.^[^ ^78^ ^]^

### Sound Velocity

Sound velocity change measurements were performed using the ultrasound option for PPMS from Quantum Design (data in Figure S9, Supporting Information). The measurements were performed using the phase comparison method.^[^ ^79^ ^]^ LiNbO_3_ transducers were glued on Thiokol LP032 glue.

## Conflict of Interest

The authors declare no conflict of interest.

## Supporting information

Supporting Information

## Data Availability

The data that support the findings of this study are available from the corresponding author upon reasonable request.
