# Supplementary material for: A Frustrated Antipolar Phase Analogous to Classical Spin Liquids
Source: Adv Mater. 2024 Oct 23;36(50):2410282. doi: 10.1002/adma.202410282 (PMC11635917; doi:10.1002/adma.202410282)
Supplement: Supplementary file 1 — Supporting Information [file ADMA-36-2410282-s001.pdf]

ADVANCED MATERIALS

Supporting Information

for *Adv. Mater.*, DOI 10.1002/adma.202410282

A Frustrated Antipolar Phase Analogous to Classical Spin Liquids

Gaël Bastien, Dalibor Repček, Adam Eliáš, Andrej Kancko, Quentin Courtade, Tetiana Haidamak, Maxim Savinov, Viktor Bovtun, Martin Kempa, Karel Carva, Michal Vališka, Petr Doležal, Marie Kratochvílová, Sarah A. Barnett, Petr Proschek, Jan Prokleška, Christelle Kadlec, Petr Kužel, Ross H. Colman and Stanislav Kamba**

A frustrated antipolar phase analogous to classical spin liquids
Supplemental information

G. Bastien,[1] D. Repček,[2,3] A. Eliáš,[1] A. Kancko,[1] Q. Courtade,[1] T. Haidamak,[1] M. Savinov,[2] V. Bovtun,[2] M. Kempa,[2] K. Carva,[1] M. Vališka,[1] P. Doležal,[1] M. Kratochvílová,[1] S. A. Barnett,[4] P. Proschek,[1] J. Prokleška,[1] C. Kadlec,[2] P. Kužel,[2] R. H. Colman,[1] and S. Kamba[2]

[1]*Charles University, Faculty of Mathematics and Physics, Department of Condensed Matter Physics, Ke Karlovu 5, 121 16 Prague 2, Czech Republic*

[2]*Institute of Physics, Czech Academy of Sciences, Na Slovance 2, 182 00 Prague, Czech Republic*

[3]*Czech Technical University in Prague, Faculty of Nuclear Sciences and Physical Engineering, Department of Solid State Engineering, Břehová 7, 115 19 Prague 1, Czech Republic*

[4]*Diamond Light Source, Chilton, Didcot, Oxfordshire, OX11 0DE, United Kingdom*

I. CRYSTAL STRUCTURE RELAXATION AND PHONON SPECTRA COMPUTATION

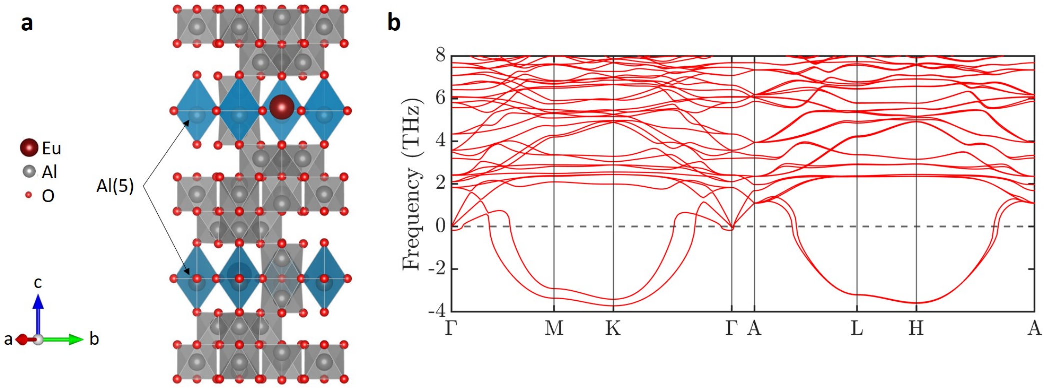

Figure S 1. **a** Schematic view of the ferroelectric crystal structure obtained upon relaxation from the experimentally determined crystal structure of $EuAl_{12}O_{19}$. The relaxation was performed on a single unit cell with periodic boundary condition. In the relaxed structure, the Al(5) ions are displaced downwards from the centre of their bipyramid. **b** Phonon dispersion structure calculated for the relaxed structure. Only phonons below 8 THz are shown to focus on the low energy branches and soft modes. Imaginary frequencies are represented by negative values.

Since DFT+U calculations performed with the magnetoplumbite-type centrosymmetric structure (space group $P6_3/mmc$) revealed its instability (see Fig. 1g in the main text), we performed a second calculation with an off-centering of Al(5) in the bipyramid AlO_5. The unit cell of the crystal structure of $EuAl_{12}O_{19}$ contains two Al(5) located in two different triangular layers of electric dipoles (Fig. 1c in the main text or Fig S5). We have performed structural relaxation both with parallel and antiparallel alignment of these two electric dipoles starting from the structure determined by x-ray diffraction, where only the Al(5) ion violates the symmetry by the mirror plane $z = 1/4$. The centrosymmetric structure without electric dipoles was also relaxed for comparison. Total calculated energies of an infinite periodic system with both ordering and with an absence of electric dipoles were compared and the ferroelectric order is the most favorable. This indicates the tendency to form electric dipoles, as well as preference for parallel alignment of dipoles located on adjacent triangular layers. A schematic view of the relaxed structure is shown in Fig. S1a and the atomic coordinates obtained after relaxation are shown in Tab SI. Upon relaxation, the Al(5) ions are displaced by $\delta = 0.25$ Å in good agreement with the experimental value of $\delta = 0.24$ Å from MEM analysis at $T = 35$ K. In addition, the relaxation indicates a vertical displacement of the O(1) ions forming the basal triangle of the bipyramid AlO_5 of $\delta_O = 0.07$ Å, which cannot be resolved from x-ray diffraction. The relaxed crystal structure belongs to the non-centrosymmetric space group $P6_3mc$.

As the next step we have evaluated phonon spectra for an infinite periodic system with the unit cell deduced from the relaxation (Fig. S1b). Two soft modes are still present, but reach imaginary

frequencies (plotted as negative values) only near points M, K, L, H; not at Γ and A. This corresponds to k-points approaching the boundary of the Brillouin zone in the planar direction. These two soft modes are dominated by the displacement of Al in the bipyramid AlO_5 in the c direction and their dispersion indicates that phonon modes with Al displaced oppositely in neighbouring cell in the ab plane would be of lowest energy. These results hint for a preference for antiparrallel alignement of the nearest neighbors as it was further confirmed by the computation of energy in a system made of two unit cells (see main text, section 2.1). It implies the presence of antipolar interactions within the triangular lattice of electric dipoles in the ab plane which is the key ingredient for the formation of geometrical frustration.

Table S I. Atomic coordinates of the relaxed crystal structure of $EuAl_{12}O_{19}$. The cell parameters were kept constant at the experimental values obtained from structural refinement of single crystal diffraction at T = 35 K, a = 5.56480 Å and c = 21.9912 Å. The left column gives the results of a relaxation with the inversion symmetry initially broken by slightly displacing Al(5) atom in the z direction resulting in a ferroelectric crystal structure (space group $P6_3mc$). The right coloumn gives the results of a relaxation performed upon starting with a centrosymmetric crystal structure (space group $P6_3/mmc$).

	Al(5) off-center			centrosymmetric structure		
Atom	x	y	z	x	y	z
Eu	0.33333	0.66667	0.75000	0.33333	0.66667	0.75000
Al(1)	0.16873	0.33747	0.60542	0.16808	0.33617	0.60789
Al(2)	0.33333	0.66667	0.18801	0.33333	0.66667	0.19034
Al(3)	0.00000	0.00000	0.99768	0.00000	0.00000	0.00000
Al(4)	0.33333	0.66667	0.02675	0.33333	0.66667	0.02909
Al(5)	0.00000	0.00000	0.23880	0.00000	0.00000	0.25000
O(1)	0.18138	0.36275	0.24690	0.18178	0.36356	0.25000
O(2)	0.50255	0.00510	0.14571	0.50213	0.00426	0.14774
O(3)	0.00000	0.00000	0.14798	0.00000	0.00000	0.14881
O(4)	0.15428	0.30859	0.05023	0.15465	0.30934	0.05169
O(5)	0.33333	0.66667	0.55179	0.33333	0.66667	0.55380

II. ELECTRIC FIELD DEPENDENCE OF THE PYROELECTRIC CURRENTS

Pyroelectric currents were measured using the common procedure for the study of ferroelectric materials [1]. A single crystal of $EuAl_{12}O_{19}$ was cooled under a bias electric field applied along the c axis down to T = 2 K. Then the electric field was removed and after a dwelling time of 15 minutes the pyroelectric current of the crystal was measured upon heating and the temperature dependence of the polarization was obtained by its integration. The polarization as a function of temperature and electric field is shown in Fig. S2. The polarization at T = 2 K shows no signs of saturation (see inset of Fig. S2), it is proportional to the electric field $P = \varepsilon_0 \varepsilon_c E$ with the value ε_c=73, which agrees rather well with the experimental value of the dielectric permittivity in Fig. 2b of the main text. Fig. S2 indicates that the polarization is induced by the applied electric field and it does not correspond to any spontaneous polarization. This electric field-induced polarization can be observed because the dynamics of the electric dipoles AlO_5 at T = 2 K are slow enough to maintain the polarization after the removal of the electric field at least on the timescale of an hour (see Fig5c-d). The relaxation of the polarization with time is evidenced by a significant decrease in polarization when heated (without field) from 2 to 20 K.

We expect the polarization of the ITLAFE to deviate from linear behavior at finite electric field and to show a saturation at $P = n\mu/3$ analogous to magnetization plateaus in triangular magnets [2, 3]. This saturation would be related to the formation of an improper ferroelectric up-up-down order (see Fig. 1**f** in the main text). Using the magnitude of the electric dipole μ = 0.97 e.Å extracted from the Curie-Weiss fit and the density of electric dipoles $n = 3.38.10^{27}$ m^{-3}, we can estimate the polarization of this phase at $P \approx 18$ mC/m^2. This value exceeds by almost two orders of magnitude the polarization P = 0.39 mC/m^2 achieved at the highest electric field applied, E = 6 kV/cm. Thus the observed linearity of the polarization in applied electric field does not show any contradiction with

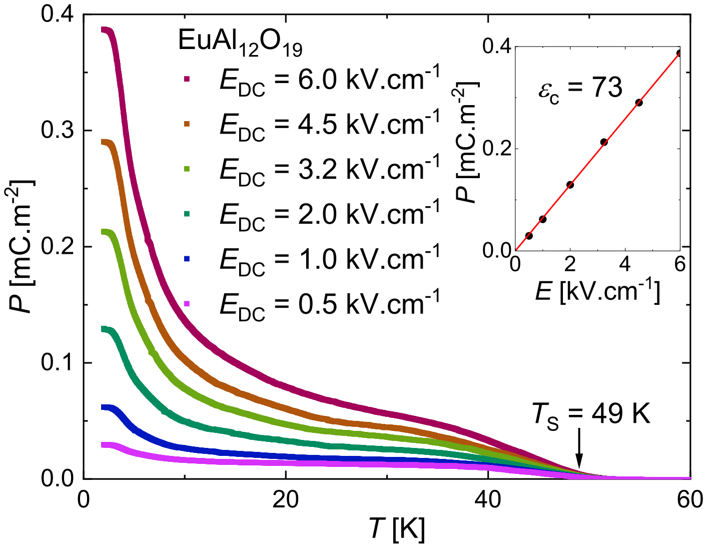

Figure S 2. Temperature dependence of polarization along the c axis calculated from pyrocurrent measurements as a function of poling electric field. The measurements were performed upon heating at a constant rate of $q = 2\,\mathrm{K/min}$ in absence of external electric field. The inset shows the polarization at $T = 2\,\mathrm{K}$ as a function of the poling field E. It confirms the assumption of a linearly induced polarization $P = \varepsilon_0 \varepsilon_c E$, with a static dielectric permittivity of $\varepsilon_c = 73$.

the expectation of a polarization plateau in the ITLAFE.

III. DIELECTRIC PERMITTIVITY UNDER APPLIED ELECTRIC BIAS FIELD

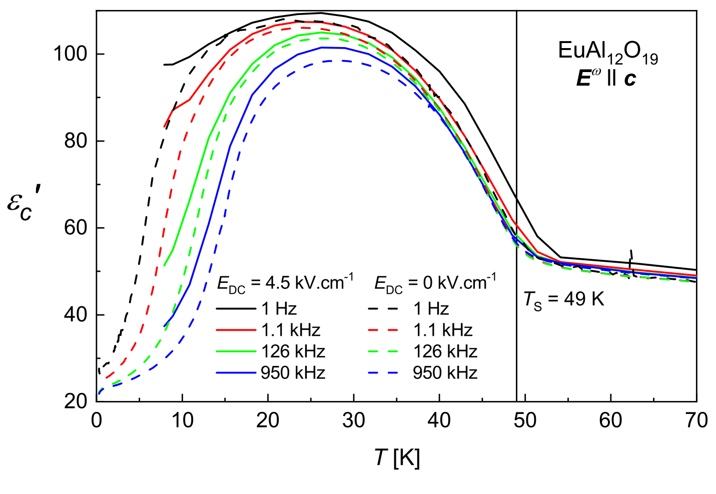

Figure S 3. Comparison of temperature dependence of the dielectric permittivity measured under bias electric field $E_{\mathrm{DC}} = 4.5\,\mathrm{kV/cm}$ (applied along the c axis) with the dielectric permittivity measured without electric field.

In ferroelectric states, the vibration of the domain walls can contribute strongly to the dielectric permittivity [4–6]. In addition, the number of domain walls is expected to decrease upon application of a static electric field E_{DC}, consequently, the dielectric response should be diminished.[4–6]. Thus measurements of the dielectric permittivity under applied static (DC) electric field E_{DC} are often used to distinguish between the contribution of domain walls and the intrinsic contribution from the bulk [4–6].

However, our experiments with $E_{DC} = 0\,kV/cm$ and $E_{DC} = 4.5\,kV/cm$ showed no significant difference in ε_c' (see Fig. S3). The observed differences are due to the different cryostats used and therefore lie within the accuracy of the measurements. The dielectric response of the relaxation R2 is thus practically the same and it cannot be ascribed to the contribution of ferroelectric domain walls. These results further support the absence of ferroelectric order.

IV. ABSENCE OF SUPERSTRUCTURE PEAKS IN THE SINGLE CRYSTAL X-RAY DIFFRACTION DATA

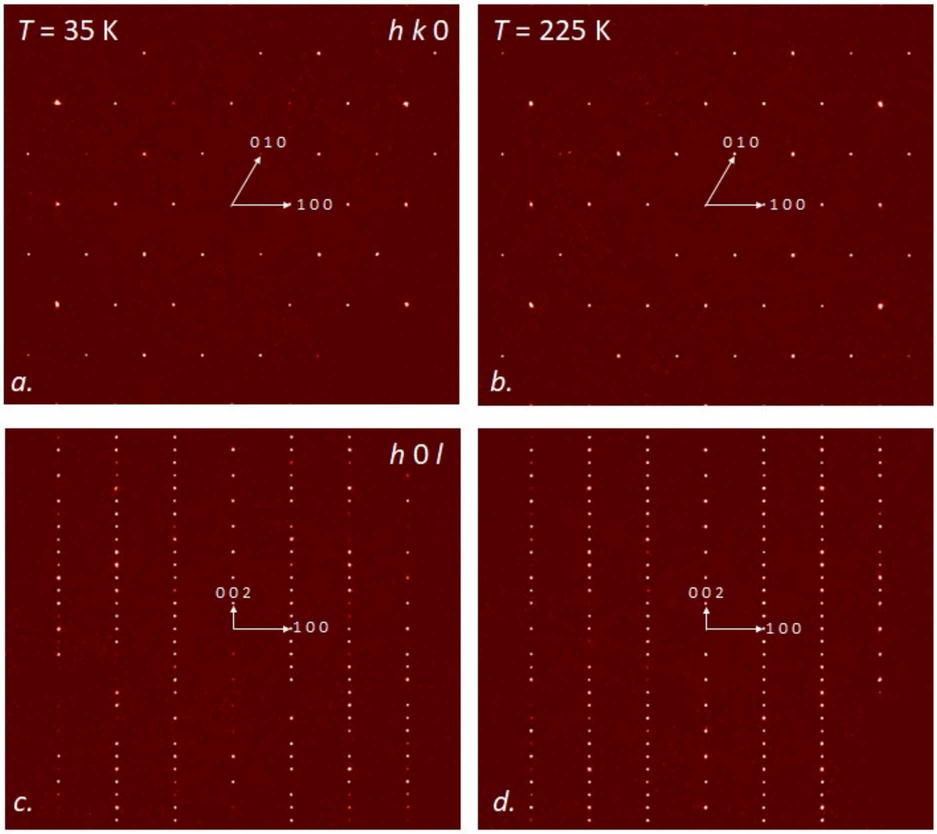

Figure S 4. Reconstructed reciprocal space maps within the $hk0$ (a and b) and $h0l$ (c and d) planes at $T = 35\,K$ and $T = 225\,K$, without absorption correction.

To further show the absence of superstructure in $EuAl_{12}O_{19}$ at $T_S = 49\,K$, we plotted reconstructed reciprocal space maps in Fig. S4 obtained at $T = 35\,K$ and $T = 225\,K$. Long-range antiferroelectric order or improper ferroelectric order with antipolar alignment of dipoles located in the same *ab* plane would imply a multiplication of the unit cell that would result in additional peaks within these reciprocal space maps. No superstructure peaks are observed at $T = 35\,K$ and their absence shows the absence of multiplication of the unit cell at $T_S = 49\,K$. This transition may still be associated with minor changes of the crystal structure beyond the resolution of the x-ray diffraction experiments, however the present measurements demonstrate that the electric dipoles AlO_5 do not form any long range order at this transition.

V. REFINEMENT DETAILS OF TEMPERATURE DEPENDENT SINGLE CRYSTAL DIFFRACTION

The detailed results from the refinement of the data from SCXRD are given in Tables SII to SV.

Table S II. Details of the single crystal structure solution of $EuAl_{12}O_{19}$ using the I19 single crystal diffractometer at the Diamond Light Source, at T = 35 K

Space group: $P6_3/mmc$ (#194, setting 1)	a = 5.56480(3) Å	c = 21.9912(2) Å	V = 589.8 Å^3	Z = 2	
Radiation: X-rays synchrotron (λ = 0.6889 Å)	Reflections collected/unique/used: 13099/725/588	Final R indices: R = 3.10 % wR_2 = 7.39 % Parameters: 42	Maximum difference: Peaks & Holes 0.56 eÅ^{-3} & −0.68 eÅ^{-3}	Density: ρ = 4.3908 g.cm^{-3}	Absorption coefficient: μ = 5.826 mm^{-1}

T (K)	Atom	Site	x	y	z	Occ.	U_{ij} ($\times10^4$Å^2) U_{11}	U_{22}	U_{33}	U_{12}	U_{13}	U_{23}	U_{eq}
35 K	Eu	2d	$\frac{1}{3}$	$\frac{2}{3}$	$\frac{3}{4}$	1	20(1)	U_{11}	25(2)	$\frac{1}{2}U_{11}$	0	0	22(1)
	Al(1)	12k	0.16834(7)	0.33668(7)	0.60833(4)	1	14(3)	U_{11}	29(4)	6(3)	$-U_{23}$	2(1)	19(3)
	Al(2)	4f	$\frac{1}{3}$	$\frac{2}{3}$	0.19073(7)	1	17(4)	U_{11}	22(6)	$\frac{1}{2}U_{11}$	0	0	18(3)
	Al(3)	2a	0	0	0	1	12(5)	U_{11}	8(7)	$\frac{1}{2}U_{11}$	0	0	11(4)
	Al(4)	4f	$\frac{1}{3}$	$\frac{2}{3}$	0.02831(7)	1	17(4)	U_{11}	21(6)	$\frac{1}{2}U_{11}$	0	0	18(3)
	Al(5)	4e	0	0	0.24015(12)	$\frac{1}{2}$	15(5)	U_{11}	30(2)	$\frac{1}{2}U_{11}$	0	0	19(8)
	O(1)	6h	0.1816(2)	0.3632(5)	$\frac{1}{4}$	1	34(8)	21(10)	39(11)	$\frac{1}{2}U_{22}$	0	0	33(7)
	O(2)	12k	0.5022(3)	0.00440(16)	0.14809(9)	1	19(7)	18(5)	26(7)	$\frac{1}{2}U_{11}$	$2U_{23}$	3(3)	21(5)
	O(3)	4e	0	0	0.14856(14)	1	12(7)	U_{11}	25(11)	$\frac{1}{2}U_{11}$	0	0	16(6)
	O(4)	12k	0.15478(17)	0.3096(3)	0.05196(9)	1	15(6)	15(7)	27(8)	$\frac{1}{2}U_{22}$	$\frac{1}{2}U_{23}$	3(5)	19(5)
	O(5)	4f	$\frac{1}{3}$	$\frac{2}{3}$	0.55440(16)	1	24(8)	U_{11}	24(13)	$\frac{1}{2}U_{11}$	0	0	24(7)

Table S III. Details of the single crystal structure solution of $EuAl_{12}O_{19}$ using the I19 single crystal diffractometer at the Diamond Light Source, at T = 80 K

Space group: $P6_3/mmc$ (#194, setting 1)	a = 5.56480(2) Å	c = 21.9788(2) Å	V = 589.4 Å^3	Z = 2	
Radiation: X-rays synchrotron (λ = 0.6889 Å)	Reflections collected/unique/used: 13284/722/590	Final R indices: R = 3.22 % wR_2 = 8.84 % Parameters: 42	Maximum difference: Peaks & Holes 0.44 eÅ^{-3} & −0.62 eÅ^{-3}	Density: ρ = 4.3933 g.cm^{-3}	Absorption coefficient: μ = 5.829 mm^{-1}

T (K)	Atom	Site	x	y	z	Occ.	U_{ij} ($\times10^4$Å^2) U_{11}	U_{22}	U_{33}	U_{12}	U_{13}	U_{23}	U_{eq}
80 K	Eu	2d	$\frac{1}{3}$	$\frac{2}{3}$	$\frac{3}{4}$	1	30(1)	U_{11}	29(2)	$\frac{1}{2}U_{11}$	0	0	30(1)
	Al(1)	12k	0.16833(4)	0.33666(4)	0.60829(5)	1	14(3)	U_{11}	16(4)	10(3)	$-U_{23}$	1(1)	14(3)
	Al(2)	4f	$\frac{1}{3}$	$\frac{2}{3}$	0.19047(7)	1	23(4)	U_{11}	10(6)	$\frac{1}{2}U_{11}$	0	0	19(3)
	Al(3)	2a	0	0	0	1	20(4)	U_{11}	2(7)	$\frac{1}{2}U_{11}$	0	0	14(4)
	Al(4)	4f	$\frac{1}{3}$	$\frac{2}{3}$	0.02838(7)	1	12(4)	U_{11}	30(8)	$\frac{1}{2}U_{11}$	0	0	18(4)
	Al(5)	4e	0	0	0.24043(12)	$\frac{1}{2}$	17(5)	U_{11}	2(20)	$\frac{1}{2}U_{11}$	0	0	12(7)
	O(1)	6h	0.18115(18)	0.3623(4)	$\frac{1}{4}$	1	28(7)	14(6)	45(9)	$\frac{1}{2}U_{22}$	0	0	31(5)
	O(2)	12k	0.50232(8)	0.00464(16)	0.14809(8)	1	28(6)	25(5)	17(6)	$\frac{1}{2}U_{11}$	$2U_{23}$	1(2)	23(4)
	O(3)	4e	0	0	0.14846(11)	1	28(6)	U_{11}	43(9)	$\frac{1}{2}U_{11}$	0	0	33(5)
	O(4)	12k	0.15490(12)	0.3098(2)	0.05170(10)	1	24(5)	15(5)	31(7)	$\frac{1}{2}U_{22}$	$\frac{1}{2}U_{23}$	5(4)	25(4)
	O(5)	4f	$\frac{1}{3}$	$\frac{2}{3}$	0.55428(16)	1	32(8)	U_{11}	16(13)	$\frac{1}{2}U_{11}$	0	0	27(7)

Table S IV. Details of the single crystal structure solution of $EuAl_{12}O_{19}$ using the I19 single crystal diffractometer at the Diamond Light Source, at $T = 120$ K

Space group: $P6_3/mmc$ (#194, setting 1)	$a = 5.56620(18)$ Å	$c = 21.9871(1)$ Å	$V = 590.0$ Å^3		$Z = 2$
Radiation: X-rays synchrotron ($\lambda = 0.6889$ Å)	Reflections collected/unique/used: 13554/725/613	Final R indices: $R = 1.77$ %, $wR_2 = 5.71$ %, Parameters: 42	Maximum difference: Peaks & Holes 0.25 eÅ^{-3} & −0.51 eÅ^{-3}	Density: $\rho = 4.3894$ g.cm^{-3}	Absorption coefficient: $\mu = 5.824$ mm^{-1}

T (K)	Atom	Site	x	y	z	Occ.	U_{ij} (×10^{4}Å^2)						
							U_{11}	U_{22}	U_{33}	U_{12}	U_{13}	U_{23}	U_{eq}
120 K	Eu	2d	$\frac{1}{3}$	$\frac{2}{3}$	$\frac{3}{4}$	1	37(1)	U_{11}	34(1)	$\frac{1}{2}U_{11}$	0	0	36(1)
	Al(1)	12k	0.16830(4)	0.33660(4)	0.60834(3)	1	21(2)	U_{11}	20(3)	11(2)	$-U_{23}$	1(1)	21(2)
	Al(2)	4f	$\frac{1}{3}$	$\frac{2}{3}$	0.19058(5)	1	25(2)	U_{11}	8(4)	$\frac{1}{2}U_{11}$	0	0	20(2)
	Al(3)	2a	0	0	0	1	21(3)	U_{11}	14(5)	$\frac{1}{2}U_{11}$	0	0	19(2)
	Al(4)	4f	$\frac{1}{3}$	$\frac{2}{3}$	0.02832(5)	1	24(3)	U_{11}	21(4)	$\frac{1}{2}U_{11}$	0	0	23(2)
	Al(5)	4e	0	0	0.24054(7)	$\frac{1}{2}$	21(3)	U_{11}	12(14)	$\frac{1}{2}U_{11}$	0	0	18(5)
	O(1)	6h	0.18132(15)	0.36260(3)	$\frac{1}{4}$	1	47(6)	13(6)	43(7)	$\frac{1}{2}U_{22}$	0	0	38(4)
	O(2)	12k	0.50250(8)	0.00499(17)	0.14809(5)	1	30(4)	25(3)	25(4)	$\frac{1}{2}U_{11}$	$2U_{23}$	1(1)	26(3)
	O(3)	4e	0	0	0.14841(8)	1	25(4)	U_{11}	22(6)	$\frac{1}{2}U_{11}$	0	0	24(4)
	O(4)	12k	0.15491(10)	0.30980(2)	0.05185(6)	1	28(4)	25(4)	22(5)	$\frac{1}{2}U_{22}$	$\frac{1}{2}U_{23}$	2(3)	25(3)
	O(5)	4f	$\frac{1}{3}$	$\frac{2}{3}$	0.55422(10)	1	30(5)	U_{11}	3(8)	$\frac{1}{2}U_{11}$	0	0	21(4)

Table S V. Details of the single crystal structure solution of $EuAl_{12}O_{19}$ using the I19 single crystal diffractometer at the Diamond Light Source, at $T = 225$ K

Space group: $P6_3/mmc$ (#194, setting 1)	$a = 5.56800(2)$ Å	$c = 21.9997(1)$ Å	$V = 590.7$ Å^3		$Z = 2$
Radiation: X-rays synchrotron ($\lambda = 0.6889$ Å)	Reflections collected/unique/used: 13176/725/621	Final R indices: $R = 1.85$ %, $wR_2 = 5.58$ %, Parameters: 42	Maximum difference: Peaks & Holes 0.74 eÅ^{-3} & −1.24 eÅ^{-3}	Density: $\rho = 4.3840$ g.cm^{-3}	Absorption coefficient: $\mu = 5.817$ mm^{-1}

T (K)	Atom	Site	x	y	z	Occ.	U_{ij} (×10^{4}Å^2)						
							U_{11}	U_{22}	U_{33}	U_{12}	U_{13}	U_{23}	U_{eq}
225 K	Eu	2d	$\frac{1}{3}$	$\frac{2}{3}$	$\frac{3}{4}$	1	51(1)	U_{11}	50(1)	$\frac{1}{2}U_{11}$	0	0	50(1)
	Al(1)	12k	0.16836(4)	0.33671(8)	0.60836(3)	1	27(2)	U_{11}	27(3)	11(1)	$-U_{23}$	3(1)	26(2)
	Al(2)	4f	$\frac{1}{3}$	$\frac{2}{3}$	0.19052(5)	1	24(2)	U_{11}	21(4)	$\frac{1}{2}U_{11}$	0	0	23(2)
	Al(3)	2a	0	0	0	1	28(3)	U_{11}	25(4)	$\frac{1}{2}U_{11}$	0	0	25(2)
	Al(4)	4f	$\frac{1}{3}$	$\frac{2}{3}$	0.02829(4)	1	25(3)	U_{11}	23(1)	$\frac{1}{2}U_{11}$	0	0	25(2)
	Al(5)	4e	0	0	0.24057(7)	$\frac{1}{2}$	26(3)	U_{11}	23(13)	$\frac{1}{2}U_{11}$	0	0	25(5)
	O(1)	6h	0.1813(3)	0.36260(15)	$\frac{1}{4}$	1	21(6)	56(6)	42(7)	$\frac{1}{2}U_{22}$	0	0	44(4)
	O(2)	12k	0.50242(9)	0.00484(9)	0.14811(5)	1	29(3)	29(3)	37(4)	$\frac{1}{2}U_{11}$	$2U_{23}$	5(1)	33(3)
	O(3)	4e	0	0	0.14831(8)	1	23(4)	U_{11}	44(7)	$\frac{1}{2}U_{11}$	0	0	30(4)
	O(4)	12k	0.1551(2)	0.31020(10)	0.05176(6)	1	16(4)	25(4)	32(5)	$\frac{1}{2}U_{22}$	$\frac{1}{2}U_{23}$	2(3)	27(3)
	O(5)	4f	$\frac{1}{3}$	$\frac{2}{3}$	0.55435(10)	1	31(5)	U_{11}	17(8)	$\frac{1}{2}U_{11}$	0	0	26(4)

VI. ELECTRON DISTRIBUTION MAP EXTRACTED BY THE MAXIMUM OF ENTROPY METHOD (MEM)

The electron distribution map was obtained by the Maximum of Entropy Method (MEM) using the Dysnomia software suite [7] and it is represented in Fig. S5 together with the crystal structure.

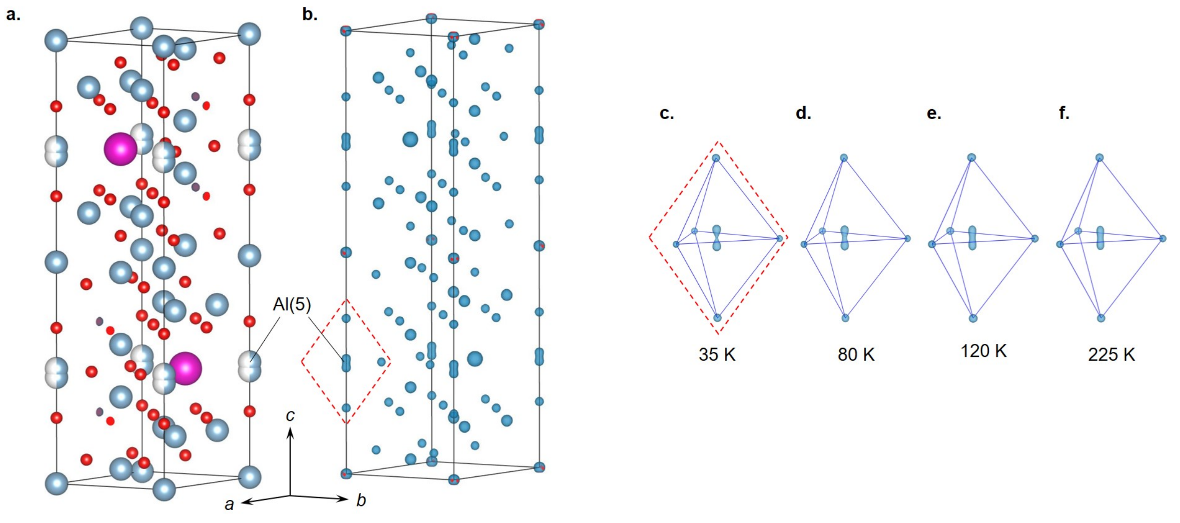

Figure S 5. Side-by-side comparison of the $EuAl_{12}O_{19}$ single crystal structure solution (a) at T = 35 K, with an isosurface plot of the MEM-calculated electron density distribution map (b) at an isosurface value of 3.5 eÅ^{-3}. The off-mirror-plane displacement of the Al(5) site is observed in the MEM-calculation in agreement with the split-site model used for the refinement. (c-f) The temperature-dependent changes of the Al(5) electron density distribution, as well as those of the surrounding bipyramid coordinating oxygen sites, with an isosurface level of 8.9 eÅ^{-3}. The apparent electron density bridging the two sites reduces on cooling from 225 K to 35 K, with no evident distortion of the oxygen atoms that create the coordination cage.

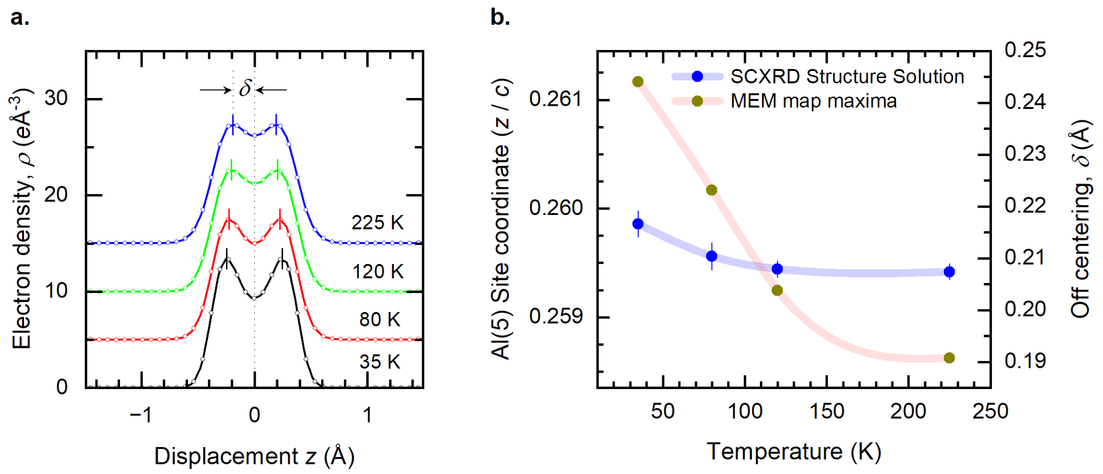

Figure S 6. a. Electron density as a one-dimensional cut along the c axis bisecting the Al(5) site (x=0, y=0, z). The curves are offset for clarity. The off-centering δ of the Al(5) ion with respect to its oxygen cage is defined as indicated in the figure as the distance between the maximum of the electron density and the center of the bipyramid. (b.) Comparison of the temperature dependence of the refined atomic z-coordinate of the Al(5) ion with the maximum in the electron density map of the MEM distribution. The right axis shows absolute values of the off-centering distance δ.

Anomalous atomic displacement is observed only for the Al(5) ions, thus it is reasonable to assume that the measured dielectric relaxations in $EuAl_{12}O_{19}$ are mainly related to this particular site.

Cuts of the electron density along the diagonal of the bipyramid AlO_5 at different temperatures are shown in Fig. S6a. They unambiguously show the splitting of the Al(5) site into two off-centered positions at all temperatures. The temperature dependence of the off-centering of the Al(5) ion δ from structure solution and from MEM cuts are represented in Fig. S6b. Both methods show an en-

hancement of the off-centering upon cooling. This evolution can be explained from the reduction of the occupancy of the center of the bipyramid upon cooling and it does not imply any evolution of the double well potential itself with temperature. Thus the double well potential can be considered as stable in temperature and the distance between the center of the bipyramid and the ground state position of the Al(5) is best estimated from the lowest temperature MEM analysis ($T = 35\,\mathrm{K}$) at $\delta \approx 0.24$. The difference between Al(5) position determined from structure solution and the maxima position within the MEM maps results from the assumptions built into single crystal solution software assuming a symmetric thermal displacement ellipsoid, which must be skewed towards the mirror plane due to additional electron density between the sites.

VII. TEMPERATURE DEPENDENCE OF LATTICE PARAMETERS

The temperature dependence of lattice parameters was determined from single crystal diffraction by following the shift of selected reflections (Fig. S7). These measurements were performed down to $T = 5\,\mathrm{K}$ using a laboratory x-ray source (see Methods section). The variation of lattice parameters and cell volume are in good agreement with thermal expansion results, especially both methods agree on the occurrence of negative thermal expansion along the c axis between $T \approx 10\,\mathrm{K}$ and $T \approx 100\,\mathrm{K}$. This negative thermal expansion along the c axis exceeds the positive thermal expansion in the ab plane implying an expansion of the crystal volume upon cooling (Fig. S7c).

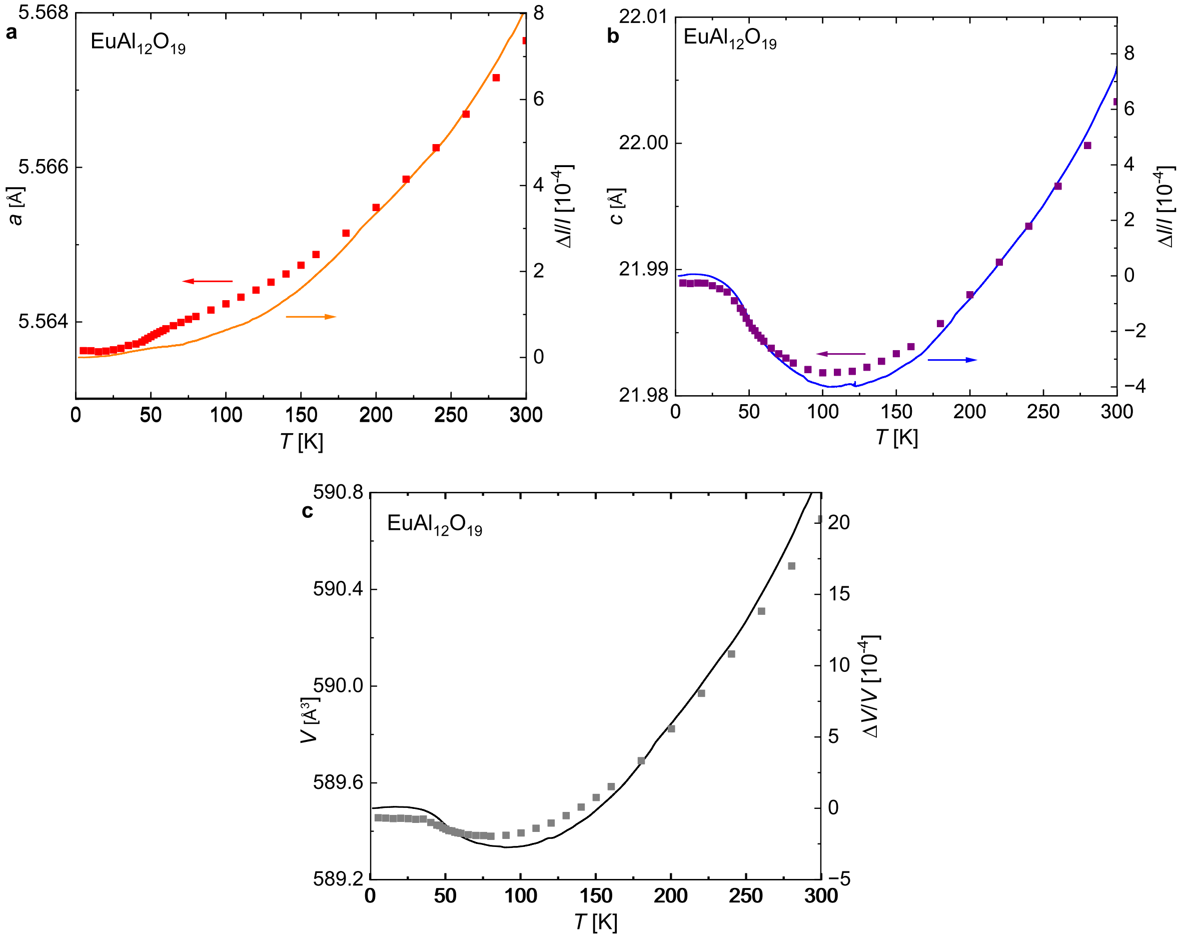

Figure S 7. Temperature dependence of the lattice parameter a in **a**, the lattice parameter c in **b** and the cell volume in **c** measured by single crystal x-ray diffraction. The relative change of lattice parameters and cell volume extracted from thermal expansion measurements are plotted together to show the agreement between both methods.

VIII. COMPLEX DIELECTRIC PERMITTIVITY IN THE THZ RANGE IN THE $\mathbf{E}^{\omega} \| \mathbf{a}$ POLARIZATION.

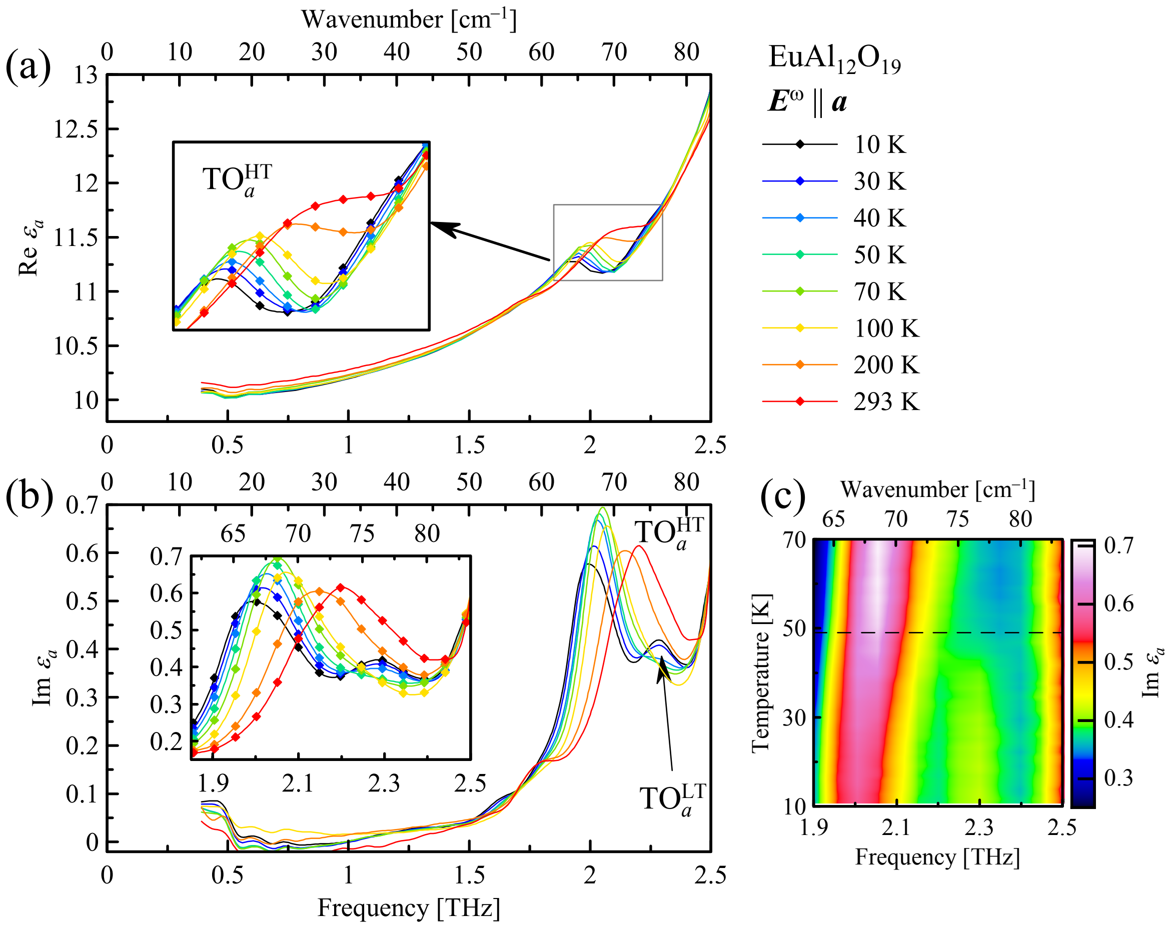

Figure S 8. Temperature evolution of the (a) real and (b) imaginary part of THz complex permittivity spectra of $EuAl_{12}O_{19}$ measured in the $\mathbf{E}^{\omega} \| \mathbf{a}$ configuration. Insets show details on TO_a^{HT} and TO_a^{LT} modes. (c) Colormap representation of the evolution of imaginary permittivity spectra between 10 and 70 K.

The spectra of THz complex permittivity directly calculated from the measured complex transmittance spectra obtained in the $\boldsymbol{E}^{\omega} \| a$ polarization complete the image of phonon dynamics in a range from $13\,\mathrm{cm}^{-1}$ (i.e. 0.4 THz) to $80\,\mathrm{cm}^{-1}$ (i.e. 2.5 THz) (Fig. S8). It does not show any sign of the dielectric relaxation R1 observed in the $\boldsymbol{E}^{\omega} \| c$ THz spectra (Fig. 4c-d in the main text) confirming the unidirectional nature of this relaxation ascribed to the Ising-like electric dipoles AlO_5. A new mode TO_a^{LT} emerges on the verge of the THz range near $76\,\mathrm{cm}^{-1}$ below T_S and thus supports the change of crystal symmetry on the local scale. Moreover, strongly anharmonic mode TO_a^{HT} dominates the spectra in the whole temperature range: it gradually softens from about $73\,\mathrm{cm}^{-1}$ at room temperature to $66\,\mathrm{cm}^{-1}$ at 10 K without any critical behavior near the phase transition at $T_S = 49$ K. In a way, it may act as a counterpart to the TO_c^{HT} phonons, which harden substantially in the $\boldsymbol{E}^{\omega} \| c$ polarized IR spectra (See Fig. 4b in the main text).

IX. CONFIRMATION OF ANISOTROPIC HARDENING UPON COOLING FROM SOUND VELOCITY EXPERIMENTS

For further insights into the origin of the phase transition at $T_S = 49\,K$ in $EuAl_{12}O_{19}$, we measured the temperature dependence of sound velocity (Fig. S9). The single crystal of $EuAl_{12}O_{19}$ belongs to hexagonal symmetry and has 5 independent elastic constants (C_{11}, C_{12}, C_{14} C_{33}, C_{44}). We focus here on the longitudinal modes C_{11} and C_{33}. The temperature dependence of the longitudinal mode

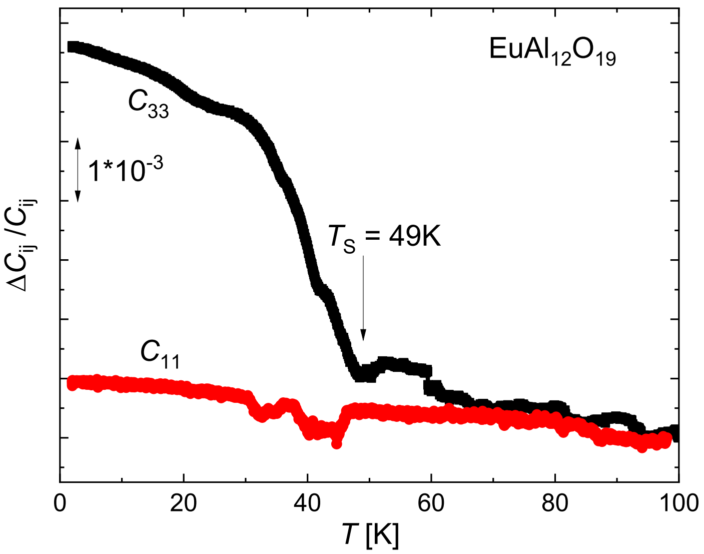

Figure S 9. Relative changes of elastic constants C_{11} and C_{33} with temperature obtained from sound velocity measurements. The phase transition at $T_S = 49\,K$ is indicated by a kink in the temperature dependence of the C_{33} mode.

along the *c* axis C_{33} shows a kink at $T_S = 49\,K$ and a hardening below this transition whereas the longitudinal mode along the *a* axis C_{11} shows a much weaker hardening. The hardening along the *c* axis can be ascribed both to structural changes associated with the phase transition at $T_S = 49\,K$ and to the change of local structure associated with the progressive formation of antipolar correlations between neighboring electric dipoles.

[1] S. Jachalke, E. Mehner, H. Stöcker, J. Hanzig, M. Sonntag, T. Weigel, T. Leisegang, and D. Meyer, How to measure the pyroelectric coefficient?, Appl. Phys. Rev. **4** (2017).

[2] K. Takasaki, I. Harada, and T. Tonegawa, Magnetic phase diagram of the Ising model on a triangular lattice with antiferromagnetic nearest-neighbor and next-nearest-neighbor interactions, J. Phys. Soc. Jpn. **55**, 4423 (1986).

[3] C. Hwang, S. Kim, D. Kang, and J. M. Kim, Thermodynamic properties of the triangular-lattice ising antiferromagnet in a uniform magnetic field, J. Korean Phys. Soc. **52**, S203 (2008).

[4] J. Kumar and A. Awasthi, Glassy domain wall matter in KH_2PO_4 crystal: Field-induced transition, Appl. Phys. Lett. **103** (2013).

[5] S. Kamba, D. Nuzhnyy, M. Savinov, P. Tolédano, V. Laguta, P. Brázda, L. Palatinus, F. Kadlec, F. Borodavka, C. Kadlec, *et al.*, Unusual ferroelectric and magnetic phases in multiferroic 2*H*-$BaMnO_3$ ceramics, Phys. Rev. B **95**, 174103 (2017).

[6] V. Goian, F. Borodavka, D. Repček, M. Savinov, M. Míšek, J. Kaštil, V. Skoromets, P. Ondrejkovič, J. Petzelt, J. Hlinka, P. Kužel, and S. Kamba, Single ferroelectric phase transition in tris-sarcosine calcium chloride, Phys. Rev. B **108**, 224101 (2023).

[7] K. Momma, T. Ikeda, A. A. Belik, and F. Izumi, Dysnomia, a computer program for maximum-entropy method (MEM) analysis and its performance in the MEM-based pattern fitting, Powder Diffraction **28**, 184 (2013).
